# The perceived control model of falling: developing a unified framework to understand and assess maladaptive fear of falling

**DOI:** 10.1093/ageing/afad093

**Published:** 2023-07-15

**Authors:** Toby J Ellmers, Mark R Wilson, Elmar C Kal, William R Young

**Affiliations:** Department of Brain Sciences, Centre for Vestibular Neurology, Imperial College London, London, UK; Centre for Cognitive Neuroscience, Brunel University London, London, UK; Department of Public Health and Sports Sciences, University of Exeter, Exeter, UK; Centre for Cognitive Neuroscience, Brunel University London, London, UK; Centre for Cognitive Neuroscience, Brunel University London, London, UK; Department of Public Health and Sports Sciences, University of Exeter, Exeter, UK

**Keywords:** concerns about falling, anxiety, balance confidence, psychology, activity avoidance, older people

## Abstract

**Background:**

fear of falling is common in older adults and can have a profound influence on a variety of behaviours that increase fall risk. However, fear of falling can also have potentially positive outcomes for certain individuals. Without progressing our understanding of mechanisms underlying these contrasting outcomes, it is difficult to clinically manage fear of falling.

**Methods:**

this paper first summarises recent findings on the topic of fear of falling, balance and fall risk—including work highlighting the protective effects of fear. Specific focus is placed on describing how fear of falling influences perceptual, cognitive and motor process in ways that might either increase or reduce fall risk. Finally, it reports the development and validation of a new clinical tool that can be used to assess the maladaptive components of fear of falling.

**Results:**

we present a new conceptual framework—the Perceived Control Model of Falling—that describes specific mechanisms through which fear of falling can influence fall risk. The key conceptual advance is the identification of perceived control over situations that threaten one’s balance as the crucial factor mediating the relationship between fear and increased fall risk. The new 4-item scale that we develop—the Updated Perceived Control over Falling Scale (UP-COF)—is a valid and reliable tool to clinically assess perceived control.

**Conclusion:**

this new conceptualisation and tool (UP-COF) allows clinicians to identify individuals for whom fear of falling is likely to increase fall risk, and target specific underlying maladaptive processes such as low perceived control.

## Key Points

- We present a new conceptual framework: the Perceived Control Model of Falling.
- This framework describes specific mechanisms through which fear of falling can increase fall risk.
- It identifies perceived control as the key factor in whether fear of falling is ultimately protective or maladaptive.
- We validate a 4-item scale—the Updated Perceived Control over Falling Scale (UP-COF)—to assess perceived control.

## Introduction

Concerns about falling are reported by up to 85% of older adults [1, 2]. They are associated with a variety of negative outcomes, including reduced physical and mental wellbeing, social isolation and increased risk for falls [1–8]. The relationship between concerns about falling and future falls was originally believed to be indirect [9]. That is, concerns about falling were thought to increase fall risk by encouraging activity restriction, which, in turn, leads to physical deconditioning and poorer balance. However, concerns about falling will often trigger an acute emotional response (i.e. ‘fear of falling’) in situations that threaten one’s balance [10, 11], leading to changes in behaviour that may *directly* increase fall risk (see Table 1 [6, 11–16]). There is, therefore, a need to conceptualise *how* and *why* this reduction in safety occurs.

**Table 1:** A description of key changes in static postural control (i.e. standing still) and walking behaviours in individuals who are fearful of falling. Note, these findings are derived from studies in which fear of falling was experimentally induced through a postural threat manipulation, rather than cross-sectional work.

***Fearful (static) postural control is...***	***Fearful walking behaviour is...***
**...cautious.** When standing still, fearful individuals will lean away from the direction of the perceived postural threat [19, 35, 36, 41, 88–90]. In general, they will also limit the amplitude and variability of swaying movements [19, 41, 88, 90], likely through an ‘ankle stiffening’ strategy (see below [19, 91]). However, very high levels of fear can actually lead to *increased* rather than reduced amplitude and variability of swaying movements [20].	**...cautious.** Fearful individuals will walk with reduced velocity, shorter steps, widened base of support and increased double-limb support (time with both feet planted on the floor) [11, 15, 92–95]. These changes increase the variability of movement (i.e. ‘stop-and-start’ and `jerky' gait [95]). Fearful individuals will also spend more time looking down at the ground for possible threats to their balance [11, 53, 76].
**...‘stiffer’.** Fearful individuals will increase the co-contraction of their lower leg and ankle muscles [35, 36, 38, 90, 91], resulting in greater frequency of swaying movements [10, 19, 35, 36, 38, 41, 43, 88–91].	**...‘stiffer’ and less fluid.** Fear of falling leads to greater activation of lower leg muscles during walking, in conjunction with reduced movement of the knee and hip joints [93].
**...consciously controlled and attentionally demanding.** Fear of falling shifts postural control from a predominately automatic [59] to a consciously controlled strategy [35, 40, 42–44, 89]. This leads to postural control becoming a more attentionally demanding process that requires attentional resources [96].	**...consciously controlled and attentionally demanding.** As with static postural control, fear of falling turns walking into a consciously controlled process [11, 53, 76], thereby increasing the attentional demands required to walk [17, 50, 71].

Building on early experimental findings [13, 15, 17–19], Hadjistavropoulos *et al.* [13] provided an initial conceptualisation of a direct relationship between fear of falling and impaired balance performance. Young and Williams [12] further developed this conceptualisation and described some specific processes through which fear of falling may disrupt balance during complex tasks and increase fall risk (e.g. via altered movement planning). Although useful, these existing frameworks do not account for: (i) why certain people experience strong and pervasive fear of falling when their balance is threatened, whereas for others the fear is mild and transient [10, 20] and (ii) why fear of falling appears to have negative impacts on fall risk for certain individuals, but potentially positive outcomes for others [6, 11, 15, 21, 22]. This understanding is necessary for effective triage and clinical management of fear of falling.

This current article describes the development of a new conceptual framework—the Perceived Control Model of Falling—designed to address these knowledge gaps. This framework consolidates empirical work and theoretical developments from research areas that are usually considered in isolation. Its key conceptual advance is the identification of perceived control over situations that threaten one’s balance as the crucial factor in whether fear of falling is ultimately protective or maladaptive with respect to fall risk. Although there is a well-established line of research linking perceived control to various outcomes of health and wellbeing in older adults [23], the present framework reflects the first attempt to formally conceptualise the link between perceived control and falls. Throughout the article, particular emphasis is placed on distinguishing the maladaptive from the protective components of fear, accounting for the paradoxical observation of why fear of falling may enhance safety in one person, yet increase fall risk in another [21, 22]. This development will allow clinicians to target the specific maladaptive components of fear, rather than attempting to indiscriminately reduce fear of falling (which could potentially do more harm than good). Finally, we present the development and validation of a new tool—the Updated Perceived Control over Falling Scale (UP-COF)—that can be used to test the model’s predictions and help guide clinical application.

### Fear and anxiety: definitions and considerations

It is first necessary to clarify the terminology used when discussing the psychological factors that influence posture, gait and fall risk. Fear of falling is often used interchangeably to refer to related—yet distinct—psychological constructs (e.g. concerns about falling, balance confidence, etc.). Without clear definitions, it is difficult to draw inferences about the extent to which these constructs affect fall risk, as they affect behaviour in different ways [24, 25].

It is important to first clarify the distinction between fear and anxiety. Although related, they are associated with unique pathologies and neural circuitry, have different physiological and behavioural correlates [26, 27], and are clinically treated via different strategies [24, 25]. It is therefore important to differentiate between the two. ‘Fear’ reflects the emotional state triggered by imminent danger; it is the awareness that one is *immediately* in harm’s way [24, 28]. In contrast, ‘anxiety’ is an emotional state triggered by an uncertain and potentially harmful event that *may or may not* occur [27, 29]. Anxiety is usually accompanied by ‘what if’ worrisome thoughts and ruminations.

Postural threats can trigger both fear and anxiety. For instance, steep stairs may first lead to the awareness that one *is* in danger of falling (fear), followed by worrisome thoughts about injuries that one *may* sustain if they *were* to fall (anxiety). Anxiety can also occur in the absence of an immediate postural threat. For instance, someone may be safe at home, but then imagine what would happen if they were to fall when leaving their house, triggering feelings of anxiety and associated autonomic symptoms. Such scenarios commonly occur in individuals with high `concerns about falling', which we define as *lasting feelings of dread and apprehension about situations that are believed to threaten or challenge balance*.

Please see Table 2 for further details on definitions regarding the psychological concepts discussed in this paper.

**Table 2:** Definitions of key terms used throughout this article.

**Term**	**Definition**
*Perceived control over falling*	One’s perceived ability to control situations that threaten or challenge balance, with respect to both the behavioural (i.e. perceived ability to control their balance and prevent a fall) and emotional response (i.e. perceived ability to harness the emotional response in helpful ways). Although linked, ‘perceived control over falling’ therefore transcends both ‘falls efficacy’ and ‘balance confidence’; as these terms simply reflect one’s perceived ability to avoid a fall, rather than one’s ability to control the threatening/challenging situation as a whole (with respect to both the behavioural and emotional response).
*Concerns about falling*	A lasting feeling of dread and apprehension about situations that are believed to threaten or challenge balance. High concerns about falling are a consequence of an individual becoming aware of their risk for experiencing an injurious fall. They lead to: (i) the increased expectation of encountering a postural threat, (ii) an inflated prediction of the potential for future harm, and (iii) a heightened emotional response (e.g. ‘fear of falling’) when balance is perceived to be threatened.
*Fear of falling*	An emotional response to a real or imagined threat to balance. Although the term ‘fear of falling’ is frequently used to describe a trait characteristic (e.g. ‘this patient *is* fearful of falling’), we contend that this is a misuse of terminology. Fear itself is a state feeling and reflects the awareness that one is in harm’s way. What is typically referred to as ‘fear of falling’ instead reflects generalised concerns about falling (see above definition).
*Anxiety*	An emotional state triggered by an uncertain and potentially harmful event that may or may not occur. Within the context of postural threats, anxiety manifests primarily as ‘what if’ worrisome thoughts about the potential consequences of falling (e.g. worries about what would happen if one were to fall and injure themselves).
*Panic response*	A sudden, uncontrollable emotional response, so strong that it overwhelms logical thought and behaviour. Leads to catastrophising (predicting solely negative outcomes, i.e. a fall) and persistent, overwhelming worrisome thoughts.
*Conscious movement processing (CMP)*	The act of directing attention internally, towards consciously planning, initiating, monitoring and/or controlling movement, with the intention of minimising motor errors or failure. Commonly triggered following an emotional response to a postural threat.

### The perceived control model of falling

**Figure 1:**
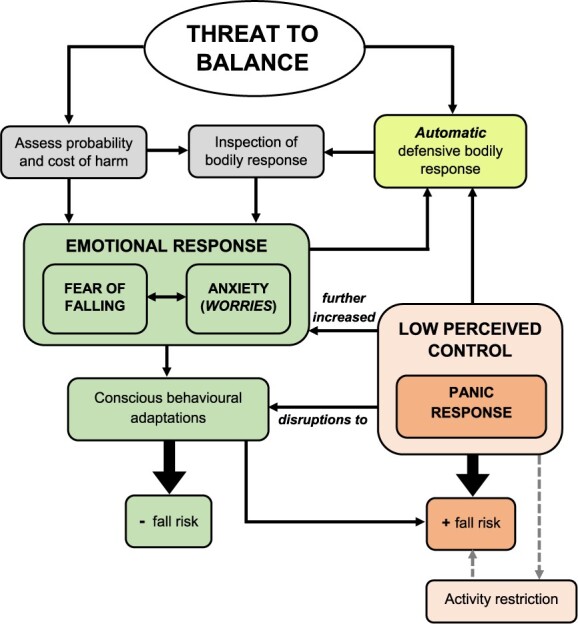
The Perceived Control Model of Falling. The central tenet of this model is that postural threats first trigger an automatic defensive bodily response (e.g. ‘postural stiffening’). Fear of falling then occurs when the individual perceives the given context as having a high probability and/or cost of harm occurring, and their appraisal of the automatic defensive response matches their ‘fear schema’ (an understanding about how one typically feels and acts when fearful of falling). This then leads to further (consciously processed) behavioural adaptations which can enhance safety. If, however, an individual has low perceived control over the given threatening context, a panic response will then be triggered, which can disrupt balance in various ways (and lead to undue activity restriction in the longer-term), thereby increasing fall risk.

The Perceived Control Model of Falling is summarised in Figure 1. It has a number of key assumptions. In line with contemporary understanding from cognitive neuroscience [24], we contend that postural threats first trigger an automatic defensive (behavioural and physiological) response. Once the postural threat has been consciously perceived, the individual will appraise the situational context (probability and cost of harm occurring) and integrate this information with their appraisal of the automatic defensive response. Fear of falling is then triggered when the individual perceives the given context as having a high probability and/or cost of harm occurring, and the automatic defensive response matches their ‘fear schema’ (an understanding about how one typically feels and acts when fearful of falling [30]). This emotional response then leads to further (consciously processed) behavioural adaptations.

The final—and perhaps most important—assumption is that some degree of fear of falling when balance is (genuinely) threatened is likely adaptive. Negative outcomes, however, arise if the emotional response triggers feelings of panic. Feelings of panic reflect an overwhelming fear response. They are the direct consequence of low perceived control over situations that threaten one’s balance, i.e. perceiving oneself as having low control over (a) preventing a fall occurring and (b) harnessing any fear experienced in helpful ways to enhance balance. Although related to existing constructs such as ‘falls efficacy’ and ‘balance confidence’, *perceived control over falling* therefore differs from these terms as it refers to an individual’s perception of control over the threatening situation as a whole (including their emotional response), rather than just their belief in their ability to avoid falling. Just as highly confident, expert athletes are susceptible to ‘choking’ in high-pressure situations because of compromised emotional regulation, older adults too will experience performance breakdown irrespective of their balance confidence/falls efficacy if they are unable to control their emotional response when balance is threatened.

The following sections will discuss the foundational aspects of the model in more detail, before outlining: (1) the proposed key role of perceived control and (2) the development and validation of a new scale designed to assess perceived control over falling.

### From threat to fear: the origins of fear of falling

We frequently experience threats to balance, e.g. when stepping out of the bath or walking across uneven ground. Although stability will be maintained via defensive behavioural responses, these adaptations occur largely subconsciously and typically without feelings of fear or anxiety. However, if someone judges the situation as one likely to cause harm, we contend that this individual would then cognitively monitor the defensive response to the postural threat [10]. If this defensive response matches the individual’s personal ‘fear schema’, then this will lead to the individual labelling the experience as such, and thus fear of falling will be experienced [30, 31]. As LeDoux and Lau [32] write, ‘You know [what you are feeling] is fear because you know what fear feels like to you’ (p. R1021).

Supported by our recent work [10], we identify high *concerns about falling* as a key factor influencing whether a postural threat triggers an emotional response. High concerns about falling are a consequence of an individual becoming aware of their risk for experiencing an (injurious) fall [6, 22, 33]. Individuals with high concerns are therefore more likely to perceive a situation as threatening, given their increased expectation of encountering a postural threat. Consequently, they interpret the situation as one where harm is highly likely to occur [29]. As outlined in Table 3, this can lead to more frequent and stronger emotional responses to perceived postural threats (i.e. ‘lowering of the threat threshold’ [10, 11]), resulting in excessive and potentially unsafe behavioural adaptations (e.g. ‘overly cautious gait’) [15] (see Table 4).

**Table 3:** A description of the mechanisms through which high concerns about falling can strengthen the emotional response to a (real or imagined) postural threat.

***How do high concerns about falling strengthen the emotional response?***
**Scenario 1. In the presence of a postural threat.** High concerns about falling lead to lasting feelings of dread and apprehension about situations that threaten or challenge balance, as well as an inflated prediction of risk for harm. This results in a state of constant ‘high-alert’, leading to the enhanced detection of postural threats. When a postural threat is perceived, these individuals will then also be more likely to: (i) interpret the situational context to indicate that harm is highly likely to occur (leading to a strong fear response; i.e. ‘lowering the threat threshold’) and; (ii) subsequently experience persistent worries about safety and/or the potential consequences of falling (leading to a strong state anxiety response; Figure 1).
**Scenario 2. Anxious (‘hypervigilant’) inspection of balance (when no imminent/immediate threat is present).** Humans frequently respond to challenges to balance without consciously processing these sensory signals. However, individuals with high concerns about falling are particularly vigilant for internal/somatic sensory signals. Such hypervigilance may result in the enhanced detection of, and subsequent misinterpretation about, ‘normal’ bodily sensations related to balance (e.g. misinterpreting a minor, inconsequential change in postural stability as signalling postural threat/imbalance). This can then lead to frequent fear of falling in the absence of a ‘genuine’ postural threat.
**Scenario 3. Thoughts and memories about falling.** Thoughts and memories about a previous fall or ‘near fall’ may cause an individual to appraise a previously neutral context in which the fall-related experience occurred as one that is likely to cause harm. This can therefore increase the likelihood that a potential postural threat will trigger an emotional response. Importantly, these memories need not necessarily concern the individual themselves; vicarious experience (e.g. memories about a friend or family member falling) can have a similar effect.

**Table 4:** A description of the key negative behavioural outcomes associated with fear of falling.

***Key negative behavioural outcomes associated with fear of falling***
**Overly cautious movement.** High concerns about falling lead to both an increased expectation of encountering a postural threat and an overestimation of harm. This results in overly cautious (consciously initiated and controlled) behavioural adaptations that often go beyond what is proportionate to maximise safety in the given context (and may even occur in the absence of any ‘genuine’ threat to balance).
**Inappropriate/unsafe motor behaviour.** Low perceptions of control when balance is threatened lead to feelings of panic. This serves to disrupt ‘adaptive’ CMP because the associated persistent worries and ruminations (e.g. “I know I am going to fall and injure myself like last time”) act like a cognitive ‘dual-task’. This in turn may disrupt the planning and regulation of adaptive CMP, as there will be less attentional resources available to consciously process ongoing movement.
**Increased distractibility for external threatening stimuli.** Low perceived control (and associated panic/worries) can impair inhibition leading to increased distractibility. This means that attention will be frequently distracted away from adaptive CMP (e.g. consciously processing the ongoing step) towards external threatening stimuli (e.g. an uneven paving stone many steps ahead in the distance). This can also lead to fearful individuals continually looking down at the floor, in order to fixate immediate threats to their balance.
**Undue activity restriction.** An overestimation of harm (i.e. high concerns about falling) coupled with low perceived control over preventing harm from occurring may also lead to undue activity restriction.

### Automatic versus conscious behavioural responses

In line with both contemporary advancements in neuroscientific theory [24, 28, 34] and recent experimental work [6, 35–37], a key assumption of the Perceived Control Model of Falling is that postural threats can trigger automatic (subcortical) responses that are distinct from the emotional experience, as well as behaviours and physiological responses related to the (conscious) emotional response. We contend that as long as the threat perceived poses a *genuine* risk to balance (see Section 7), automatic defensive responses will likely serve some degree of adaptive purpose. For instance, some level of postural ‘stiffening’ (i.e. co-contraction of ankle muscles and associated increase in postural sway frequency [35, 38]) may enhance stability when balance is threatened—particularly during situations that do not require rapid stepping responses [12]. In contrast, it is the behaviours associated with the conscious emotional experience—particularly when fear and anxiety are high—which are likely to be maladaptive (i.e. excessive/inappropriate for the given context). For instance, high fear of falling can further amplify postural ‘stiffening’ behaviours (i.e. greater increases in high-frequency postural movements [10, 20]) to levels that may compromise balance performance [20]. As described below, conscious movement processing (CMP) may be beneficial in constraining potentially maladaptive fear- and anxiety-related behavioural outcomes.

## CMP: friend or foe?

A second key assumption of the Perceived Control Model of Falling is that although fear and anxiety about falling will trigger conscious attention towards regulating balance, CMP itself is not inherently maladaptive—contrary to popular belief [39].

### Aspects of CMP that can enhance balance performance

It is well accepted that both young and older adults will direct conscious attention towards monitoring and controlling movement when balance is threatened [11, 37, 38, 40–43]. Research has reported that CMP may lead to motor and cognitive inefficiencies during both standing [44–47] and walking [11, 48–57], as it is attentionally demanding and leads to slower movements. Despite this, we argue that when balance is (genuinely) threatened, and fear and anxiety about falling are high, CMP may primarily reflect an adaptive, self-regulatory process [6].

Age-related decline in the automatic processing of posture and gait necessitates some degree of CMP [47], with the amount of CMP required for safe and successful performance increasing in-line with the level of challenge/threat [58]. Recent qualitative research revealed that older people will engage in CMP when their balance is threatened, as this allows them to maintain concentration on the task at hand and ensure that the correct motor pattern necessary for maximising safety is (consciously) planned, engaged and successfully implemented [6]. These findings support lab-based research reporting that greater neural pre-frontal cortex activation (believed to reflect CMP [59]) during movement preparation was associated with preserved movement quality in older adults during challenging walking tasks [58]. Relatedly, when fearful of falling, older adults will consciously monitor ongoing stepping movements [11, 53]—a strategy that may enhance their ability to make rapid refinements to an ongoing step [60]. This likely explains why fearful older adults are better able to adjust their steps to avoid an obstacle that suddenly appears in their path [61].

Based on the above, we propose that CMP may be crucial for ‘top-down intention-directed attention’ [62] when balance is threatened, ensuring that the performer (i) avoids distraction, (ii) engages the appropriate motor strategy required to maximise safety when their balance is threatened, and (iii) consciously monitors the ongoing movement to confirm that these strategies have been implemented as intended (allowing for further conscious adaptations as necessary).

Emerging research suggests another potentially adaptive purpose of CMP: constraining ‘unhelpful’ fear-related responses [43, 44]. For instance, although fear of falling has been shown to reliably lead to some degree of ‘postural stiffening’ (increase in high-frequency postural movement and co-contraction of lower leg muscles) [38], excessive levels of stiffening may compromise rather than enhance balance performance [12, 35]. Recent work demonstrates that CMP may serve to constrain fear-related increases in stiffening behaviours in both older [43] and young adults [63]. This implies that CMP may keep fear-related responses such as postural stiffening ‘in-check’ and prevent these from increasing to maladaptive levels. This supports work from cognitive psychology, which describes how the conscious mind can be used to ‘override’ unhelpful automatic responses [64].

### Overly cautious movement: CMP is a double-edged sword

Although we argue above for several benefits of CMP when fearful about falling, CMP can lead to negative outcomes if the motor strategy initiated is inappropriate for the current context. A common example of this is the frequently observed ‘overly-cautious gait’ [65, 66]. As noted previously, individuals with high concerns about falling have (i) an increased expectation of encountering a postural threat and (ii) an over-estimation that harm (i.e. a fall) will occur once they perceive their balance to be threatened. This can lead to overly cautious behavioural adaptations beyond what is proportionate to maintain balance in the given context [15]. However, we contend that in such situations, it is not CMP per se that is negative, but rather the inappropriate initiation of CMP due to an overestimation of harm in a relatively low-threat context.

### Low perceived control: the key maladaptive process

A major strength of the Perceived Control Model of Falling is its ability to account for the observation that fear of falling may enhance safety in one person, yet increase fall risk in another [11, 21, 22]. Drawing on previous findings [6, 67], we posit that whether an emotional response to a postural threat is ultimately adaptive or maladaptive is determined by whether the individual perceives themselves as having control over the given threatening situation as a whole (including both their behavioural and emotional response).

We propose that when perceived control is high, fear of falling will trigger CMP, which leads to conservative behavioural adaptations likely to enhance safety. However, when perceived control is low, fear of falling triggers feelings of panic, leading to catastrophising and worrisome thoughts that persist during the task itself (e.g. ruminations about previous falls [6, 68]). As with previous researchers, we view these unhelpful anxious thoughts as a key driver of maladaptive outcomes associated with fear of falling [69]: they act as a ‘dual-task’ and serve to disrupt the effective use of CMP. Low perceived control will also further enhance the initial emotional response, leading to excessive fear of falling [20]. As described in the following subsections, low perceived control thus increases fall risk in numerous ways.

#### Inappropriate/unsafe motor behaviour

Postural threats will trigger feelings of panic in individuals with low perceived control, which leads to catastrophising and persistent worrisome thoughts [6]. Processing these worrisome thoughts therefore acts like a cognitive ‘dual-task’ [70] and reduces the attentional resources available [17, 50, 71] for the effective planning, initiation and monitoring of CMP-related movement strategies [50, 58, 59]. For instance, a reduction in available attentional resources has been shown to disrupt movement planning [51] and lead to the selection of risky, unsafe behaviours [72]. Attentional resources are also required for rapid and accurate reactive stepping responses following a loss of balance [73]. Persistent worries will therefore limit the resources available for these important processes. We believe that this accounts for previous observations of disrupted movement planning and subsequently greater stepping errors in a group of fearful older adults who reported experiencing worrisome thoughts [11].

#### Increased distractibility for external threatening stimuli

Safe locomotion requires effective visual search behaviour [74, 75]. Vision is used to both look ahead to plan future stepping actions, as well as to guide and adjust the ongoing step. Fear of falling can disrupt both processes. It is well established that certain older adults will display a gaze bias towards threats to balance when fearful of falling [11, 12, 14, 16]. This can lead to individuals freezing their gaze towards the immediate/salient threat to balance (e.g. continually looking down towards their feet), at the expense of planning future stepping actions [11, 12, 14, 16, 76]. This threat-related gaze bias can also lead to individuals prematurely transferring their gaze away from an ongoing step (e.g. before the step over an obstacle has been completed), to fixate on the next upcoming environmental threat [14, 16, 76]. Such premature transfer of gaze is causally associated with increased stepping errors [77], given that this impairs one’s ability to (consciously) guide and adjust the ongoing step (as described earlier).

We posit that these maladaptive gaze behaviours are primarily a consequence of low perceived control: processing worrisome thoughts has also been shown to impair cognitive inhibition [70]. This means that individuals with low perceived control will be less able to inhibit attention from being distracted towards threatening stimuli associated with their worrisome thoughts (see [70, 78]).

#### Undue activity restriction

Activity restriction is common in older adults who regularly experience fear of falling [1]. This can trigger a debilitating spiral of physical deconditioning, falls, social isolation and a loss of one’s sense of self [3–8]. Based on our recent qualitative findings [6], we propose that high concerns about falling (i.e. an inflated prediction of harm) coupled with low perceived control over preventing harm from occurring will be a risk factor for individuals developing undue fear-related activity restriction. Supporting this notion, recent work identifies low perceived control as a barrier to physical activity in older adults post hip fracture [79]. Relatedly, fall-related catastrophising—a key hypothesised outcome of low perceived control—has been shown to predict fear-related activity restriction in both community-dwelling older adults [80] and individuals with Parkinson’s Disease [81].

### Developing the Updated Perceived Control over Falling Scale (UP-COF)

The previous sections highlight the importance of perceived control in determining whether an emotional response to a postural threat is ultimately adaptive or maladaptive. It is therefore clinically important that we have instruments to assess (1) perceived control over both preventing a fall occurring and harnessing any fear experienced in positive ways and (2) the occurrence of associated maladaptive emotional responses (e.g. panic and subsequent persistent worrisome thoughts). The following section describes the development and validation of a new clinical scale designed for this purpose.

#### Scale development

An initial 4-item Perceived Control over Falling Scale was developed by Lawrence and colleagues in 1998 [82]. However, this scale has not yet been formally validated, nor was it developed through input with older people themselves. Also, as this scale is 25 years old, it does not integrate recent developments in our understanding of fear of falling, perceived control and other associated constructs (e.g. panic). We therefore sought to modify, update and subsequently validate this existing scale to account for these limitations. Although the Perceived Control Model of Falling focuses on perceptions of control within a given threatening and potentially fear-evoking context, we sought to develop a simple clinical tool to assess recent *generalised* perceived control over falling. This allows us to circumvent the complexities and limitations of asking a patient to imagine a specific threatening context and then provide a score according to this hypothetical scenario ‘as if they were there’. Much in the same way that generalised anxiety affects situation-specific anxiety/fear about falling during threatening contexts (e.g. Sturnieks *et al*. [83]), we too contend that generalised perceived control over falling will largely map onto situation-specific perceived control.

We conducted discussions with eight older adults (with a range of balance problems and fear of falling) and three experienced clinicians who work in rehabilitation and falls-prevention services. We first presented the original Perceived Control over Falling Scale. This revealed consistent confusion and problems with interpretation for two of the four items, one of which was edited, and the other removed altogether. Three new items were then developed based on discussions with the panel, and our recent qualitative study on perceived control over falling [6]. All items were iteratively refined through further feedback from older adults and clinicians until no further issues were identified and consensus was reached on the items.

This process resulted in a 6-item UP-COF used for validation.

#### Scale validation: methods

Community-dwelling older adults (*n* = 209; mean age = 75.5; range = 60–90 years; males = 18.7%) were recruited from social support networks within the UK. All participants were free from any diagnosed progressive neurological disorder or dementia. Ethical approval was obtained from the local ethics committee, and all participants provided written informed consent.

We validated the scale following established recommendations [84], including evaluation at item-level, factor analysis, and assessment of test–retest reliability and concurrent validity of total UP-COF scores—followed by ROC analysis to determine cut-offs. Please see the Supplementary Materials for further information.

#### Scale validation: results

Following an Exploratory Factor Analysis, the scale was further refined to four items. All items of the 4-item UP-COF loaded onto a single factor. The 4-item UP-COF had both good internal consistency (Cronbach’s alpha = 0.751) and test–retest reliability (ICC = 0.718). The standard error of the measurement (SEM) was 1.5, whereas the minimal detectable difference was 0.54 on group level and 4.1 on individual level. Average score on the 4-item UP-COF was 15.9/20 (SD = 3.2; range = 0–20). ROC analyses indicated that total scores of 13/20 or below can be interpreted as low perceived control over falling: 21.2% of our sample who reported experiencing fear of falling to some degree in daily life met this threshold. Total UP-COF scores were significantly (negatively) correlated with short Falls Efficacy Scale-International [85] scores (*r* = −.567, 95%CI = [−.653, −.467], *P* < 0.001) and HADS-anxiety [86] scores (*r* = −.410, 95%CI = [−.518, −.291], *P* < 0.001).

UP-COF scores were significantly lower in individuals who had fallen repeatedly in the past 12 months (*M* = 12.9, *SD* = 4.68) compared with both non-fallers (*M* = 16.4, *SD* = 2.84, *P* < 0.001) and those who had fallen once (*M* = 16.2, *SD* = 2.61, *P* = 0.002). Scores were also significantly lower in individuals who reported that they avoided activities due to fear of falling (*M* = 14.0, *SD* = 3.75), compared with those who did not (*M* = 16.7, *SD* = 2.60, *P* < 0.001).

**Figure 2:**
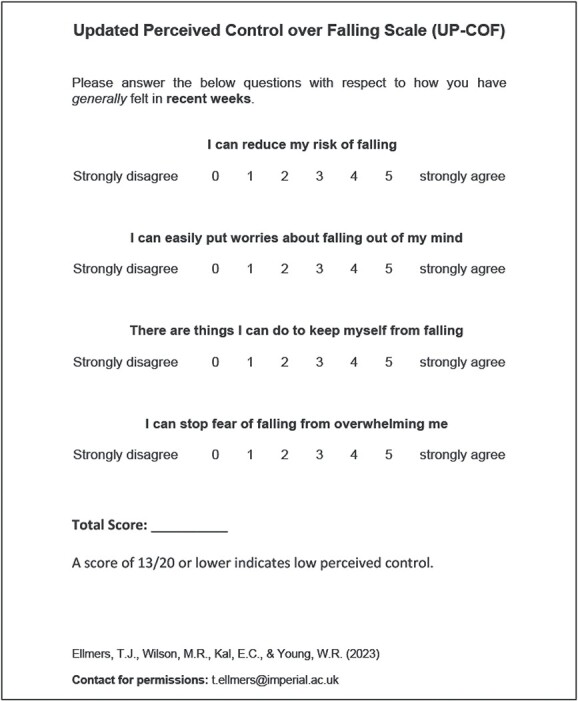
The final and validated version of the Updated Perceived Control over Falling Scale (UP-COF).

Please see the Supplementary Materials (Tables S1, S2 and S3, and Figure S1) for full results. The final validated and formatted UP-COF is presented in Figure 2.

### Summary and clinical recommendations

We present a new conceptual framework—the Perceived Control Model of Falling—that describes specific mechanisms through which fear of falling can increase fall risk. The model generates a number of key hypotheses, which should be directly tested in future experimental work (e.g. manipulating perceived control during conditions of postural threat). This new framework allows clinicians to identify individuals for whom fear of falling is likely to increase fall risk and to target specific maladaptive processes (e.g. low perceived control and associated cognitive changes). This advancement facilitates the development of new strategies to clinically manage fear of falling.

We therefore make the following clinical recommendations:

- **Assess perceived control over falling.** This can be easily achieved using the validated 4-item UP-COF scale, which will help identify individuals for whom fear of falling is likely to trigger maladaptive processes that can directly increase fall risk.
- **If perceived control is low (score ≤ 13/20):**

1. Follow-up on with more specific questions to identify specific situations/contexts in which low perceived control is most likely to manifest, and the causes for this.
2. Intervene to address the root-cause of low perceived control through both psychological and physical strategies. For instance, someone with low perceived control due to unpredictable bouts of acute instability/dizziness could be provided with strategies to enhance balance and safety during such occurrences. Increasing perceptions of control will help prevent fear of falling triggering a panic response and associated maladaptive outcomes.
3. Address the key cognitive processes associated with a panic response; namely, catastrophising (i.e. imagining the worst) and ruminations about previous falls. This can be achieved through targeted cognitive behavioural therapy strategies (e.g. Zijlstra *et al*. [87]).

## Supplementary Material

Supplementary_Materials_(1)_afad093

aa-22-1885-File003_afad093

## Data Availability

Data and analysis scripts are available via an Open Science Framework repository (https://osf.io/ghc5p/).

## References

[1] da Costa EM, Pepersack T, Godin I, Bantuelle M, Petit B, Levêque A. Fear of falling and associated activity restriction in older people. Results of a cross-sectional study conducted in a Belgian town. Arch Public Heal 2012; 70: 1. 10.1186/0778-7367-70-1 PMC3415108 22958732

[2] Scheffer AC, Schuurmans MJ, Van Dijk N, Van Der Hooft T, De Rooij SE. Fear of falling: measurement strategy, prevalence, risk factors and consequences among older persons. Age Ageing 2008; 37: 19–24. 18194967 10.1093/ageing/afm169

[3] Deshpande N, Metter EJ, Lauretani F, Bandinelli S, Guralnik J, Ferrucci L. Activity restriction induced by fear of falling and objective and subjective measures of physical function: a prospective cohort study. J Am Geriatr Soc 2008; 56: 615–20. 18312314 10.1111/j.1532-5415.2007.01639.x PMC2645621

[4] Cumming RG, Salkeld G, Thomas M, Szonyi G. Prospective study of the impact of fear of falling on activities of daily living, SF-36 scores, and nursing home admission. J Gerontol A Biol Sci Med Sci 2000; 55: M299–305. 10819321 10.1093/gerona/55.5.m299

[5] Delbaere K, Crombez G, Vanderstraeten G, Willems T, Cambier D. Fear-related avoidance of activities, falls and physical frailty. A prospective community-based cohort study. Age Ageing 2004; 33: 368–73. 15047574 10.1093/ageing/afh106

[6] Ellmers TJ, Wilson MR, Norris M, Young WR. Protective or harmful? A qualitative exploration of older people’s perceptions of worries about falling. Age Ageing 2022; 51. 10.1093/ageing/afac067. PMC8972997 35363253

[7] Hajek A, König HH. The association of falls with loneliness and social exclusion: evidence from the DEAS German ageing survey. BMC Geriatr 2017; 17: 204. 10.1186/s12877-017-0602-5. 28874139 PMC5584003

[8] Landers MR, Oscar S, Sasaoka J, Vaughn K. Balance confidence and fear of falling avoidance behavior are most predictive of falling in older adults: prospective analysis. Phys Ther 2016; 96: 433–42. 26294679 10.2522/ptj.20150184

[9] Brummel-Smith K . Falls in the aged. Prim Care 1989; 16: 377–93. 2664838

[10] Ellmers TJ, Wilson MR, Kal EC, Young WR. Standing up to threats: translating the two-system model of fear to balance control in older adults. Exp Gerontol 2022; 158: 111647. 10.1016/j.exger.2021.111647. 34861355

[11] Ellmers TJ, Cocks AJ, Young WR. Evidence of a link between fall-related anxiety and high-risk patterns of visual search in older adults during adaptive locomotion. J Gerontol A Biol Sci Med Sci 2020; 75: 961–7. 31362302 10.1093/gerona/glz176 PMC7164535

[12] Young WR, Williams AM. How fear of falling can increase fall-risk in older adults: applying psychological theory to practical observations. Gait Posture 2015; 41: 7–12. 25278464 10.1016/j.gaitpost.2014.09.006

[13] Hadjistavropoulos T, Delbaere K, Fitzgerald TD. Reconceptualizing the role of fear of falling and balance confidence in fall risk. J Aging Health 2011; 23: 3–23. 20852012 10.1177/0898264310378039

[14] Young WR, Wing AM, Hollands MA. Influences of state anxiety on gaze behavior and stepping accuracy in older adults during adaptive locomotion. J Gerontol B Psychol Sci Soc Sci 2012; 67B: 43–51. 10.1093/geronb/gbr074 21808071

[15] Delbaere K, Sturnieks DL, Crombez G, Lord SR. Concern about falls elicits changes in gait parameters in conditions of postural threat in older people. J Gerontol A Biol Sci Med Sci 2009; 64A: 237–42. 10.1093/gerona/gln014 PMC2655012 19196645

[16] Young WR, Hollands MA. Newly acquired fear of falling leads to altered eye movement patterns and reduced stepping safety: a case study. PLoS One 2012; 7. 10.1371/journal.pone.0049765. PMC3504096 23185432

[17] Gage WH, Sleik RJ, Polych MA, NC MK, Brown LA. The allocation of attention during locomotion is altered by anxiety. Exp Brain Res 2003; 150: 385–94. 12707746 10.1007/s00221-003-1468-7

[18] Adkin AL, Frank JS, Carpenter MG, Peysar GW. Fear of falling modifies anticipatory postural control. Exp Brain Res 2002; 143: 160–70. 11880892 10.1007/s00221-001-0974-8

[19] Carpenter MG, Frank JS, Silcher CP, Peysar GW. The influence of postural threat on the control of upright stance. Exp Brain Res 2001; 138: 210–8. 11417462 10.1007/s002210100681

[20] Davis JR, Campbell AD, Adkin AL, Carpenter MG. The relationship between fear of falling and human postural control. Gait Posture 2009; 29: 275–9. 18963992 10.1016/j.gaitpost.2008.09.006

[21] Litwin H, Erlich B, Dunsky A. The complex association between fear of falling and mobility limitation in relation to late-life falls: a SHARE-based analysis. J Aging Health 2018; 30: 987–1008. 28553817 10.1177/0898264317704096 PMC6655432

[22] Delbaere K, Close JCT, Brodaty H, Sachdev P, Lord SR. Determinants of disparities between perceived and physiological risk of falling among elderly people: cohort study. BMJ 2010; 341: c4165. 10.1136/bmj.c4165. 20724399 PMC2930273

[23] Robinson SA, Lachman ME. Perceived control and aging: a mini-review and directions for future research. Gerontology 2017; 63: 435–42. 28391279 10.1159/000468540 PMC5561527

[24] LeDoux JE, Pine DS. Using neuroscience to help understand fear and anxiety: a two-system framework. Am J Psychiatry 2016; 173: 1083–93. 27609244 10.1176/appi.ajp.2016.16030353

[25] Steimer T . The biology of fear- and anxiety-related behaviors. Dialogues Clin Neurosci 2002; 4: 231–49. 22033741 10.31887/DCNS.2002.4.3/tsteimer PMC3181681

[26] Davis M . Are different parts of the extended amygdala involved in fear versus anxiety? Biol Psychiatry 1998; 44: 1239–47. 9861467 10.1016/s0006-3223(98)00288-1

[27] Robinson OJ, Pike AC, Cornwell B, Grillon C. The translational neural circuitry of anxiety. J Neurol Neurosurg Psychiatry 2019; 90: 1353–60. 31256001 10.1136/jnnp-2019-321400

[28] LeDoux JE . Coming to terms with fear. Proc Natl Acad Sci U S A 2014; 111: 2871–8. 24501122 10.1073/pnas.1400335111 PMC3939902

[29] Grupe DW, Nitschke JB. Uncertainty and anticipation in anxiety: an integrated neurobiological and psychological perspective. Nat Rev Neurosci 2013; 14: 488–501. 23783199 10.1038/nrn3524 PMC4276319

[30] Izard CE . Basic emotions, natural kinds, emotion schemas, and a new paradigm. Perspect Psychol Sci 2007; 2: 260–80. 26151969 10.1111/j.1745-6916.2007.00044.x

[31] Mobbs D, Adolphs R, Fanselow MS et al. Viewpoints: approaches to defining and investigating fear. Nat Neurosci 2019; 22: 1205–16. 31332374 10.1038/s41593-019-0456-6 PMC6943931

[32] LeDoux JE, Lau H. Seeing consciousness through the lens of memory. Curr Biol 2020; 30: R1018–22. 32961150 10.1016/j.cub.2020.08.008

[33] Delbaere K, Close JCT, Mikolaizak AS, Sachdev PS, Brodaty H, Lord SR. The falls efficacy scale international (FES-I). A comprehensive longitudinal validation study. Age Ageing 2010; 39: 210–6. 20061508 10.1093/ageing/afp225

[34] LeDoux JE . The slippery slope of fear. Trends Cogn Sci 2013; 17: 155–6. 23477951 10.1016/j.tics.2013.02.004

[35] Zaback M, Adkin AL, Carpenter MG. Adaptation of emotional state and standing balance parameters following repeated exposure to height-induced postural threat. Sci Rep 2019; 9: 1–12. 31462652 10.1038/s41598-019-48722-z PMC6713771

[36] Zaback M, Luu MJ, Adkin AL, Carpenter MG. Selective preservation of changes to standing balance control despite psychological and autonomic habituation to a postural threat. Sci Rep 2021; 11: 384. 10.1038/s41598-020-79417-5. 33431937 PMC7801693

[37] Johnson KJ, Zaback M, Tokuno CD, Carpenter MG, Adkin AL. Repeated exposure to the threat of perturbation induces emotional, cognitive, and postural adaptations in young and older adults. Exp Gerontol 2019; 122: 109–15. 31028840 10.1016/j.exger.2019.04.015

[38] Adkin AL, Carpenter MG. New insights on emotional contributions to human postural control. Front Neurol 2018; 9: 789. 10.3389/fneur.2018.00789. 30298048 PMC6160553

[39] Masters R, Maxwell J. The theory of reinvestment. Int Rev Sport Exerc Psychol 2008; 1: 160–83.

[40] Huffman JL, Horslen BC, Carpenter MG, Adkin AL. Does increased postural threat lead to more conscious control of posture? Gait Posture 2009; 30: 528–32. 19729308 10.1016/j.gaitpost.2009.08.001

[41] Zaback M, Cleworth TW, Carpenter MG, Adkin AL. Personality traits and individual differences predict threat-induced changes in postural control. Hum Mov Sci 2015; 40: 393–409. 25687665 10.1016/j.humov.2015.01.015

[42] Zaback M, Carpenter MG, Adkin AL. Threat-induced changes in attention during tests of static and anticipatory postural control. Gait Posture 2016; 45: 19–24. 26979877 10.1016/j.gaitpost.2015.12.033

[43] Ellmers TJ, Kal EC, Young WR. Consciously processing balance leads to distorted perceptions of instability in older adults. J Neurol 2021; 268: 1374–84. 33141249 10.1007/s00415-020-10288-6 PMC7990754

[44] Kal EC, Young WR, Ellmers TJ. Balance capacity influences the effects of conscious movement processing on postural control in older adults. Hum Mov Sci 2022; 82: 102933. 10.1016/j.humov.2022.102933. 35134657

[45] Chow VWK, Ellmers TJ, Young WR, Mak TCT, Wong TWL. Revisiting the relationship between internal focus and balance control in young and older adults. Front Neurol 2019; 9: e1131. 10.3389/fneur.2018.01131. PMC6333651 30687212

[46] Wulf G . Attentional focus and motor learning: a review of 15 years. Int Rev Sport Exerc Psychol 2013; 6: 77–104.

[47] Boisgontier MP, Beets IAM, Duysens J, Nieuwboer A, Krampe RT, Swinnen SP. Age-related differences in attentional cost associated with postural dual tasks: increased recruitment of generic cognitive resources in older adults. Neurosci Biobehav Rev 2013; 37: 1824–37. 23911924 10.1016/j.neubiorev.2013.07.014

[48] Mak TCT, Young WR, Chan DCL, Wong TWL. Gait stability in older adults during level-ground walking: the attentional focus approach. J Gerontol B Psychol Sci Soc Sci 2020; 75: 274–81. 30299520 10.1093/geronb/gby115

[49] Uiga L, Capio CM, Ryu D et al. The role of movement-specific reinvestment in visuomotor control of walking by older adults. J Gerontol B Psychol Sci Soc Sci 2020; 75: 282–92. 29939343 10.1093/geronb/gby078

[50] Ellmers TJ, Young WR. Conscious motor control impairs attentional processing efficiency during precision stepping. Gait Posture 2018; 63: 58–62. 29715607 10.1016/j.gaitpost.2018.04.033

[51] Ellmers TJ, Cocks AJ, Doumas M, Williams AM, Young WR. Gazing into thin air: the dual-task costs of movement planning and execution during adaptive gait. PloS One 2016; 11: 1–20. 10.1371/journal.pone.0166063 PMC5100909 27824937

[52] Wong WL, Masters RSW, Maxwell JP, Abernethy AB. Reinvestment and falls in community-dwelling older adults. Neurorehabil Neural Repair 2008; 22: 410–4. 18334603 10.1177/1545968307313510

[53] Ellmers TJ, Cocks AJ, Kal EC, Young WR. Conscious movement processing, fall-related anxiety, and the visuomotor control of locomotion in older adults. J Gerontol B Psychol Sci Soc Sci 2020; 75: 1911–20. 32761087 10.1093/geronb/gbaa081 PMC7566972

[54] Young WR, Ellmers TJ, Kinrade NP, Cossar J, Cocks AJ. Re-evaluating the measurement and influence of conscious movement processing on gait performance in older adults: development of the gait-specific attentional profile. Gait Posture 2020; 81: 73–7. 32683216 10.1016/j.gaitpost.2020.07.008

[55] Ellmers TJ, Kal EC, Richardson JK, Young WR. Short-latency inhibition mitigates the relationship between conscious movement processing and overly cautious gait. Age Ageing 2021; 50: 830–7. 33951155 10.1093/ageing/afaa230 PMC8099234

[56] Uiga L, Capio CM, Wong TWL, Wilson MR, Masters RSW. Movement specific reinvestment and allocation of attention by older adults during walking. Cogn Process 2015; 16: 421–4. 26233524 10.1007/s10339-015-0685-x

[57] Young WR, Olonilua M, Masters RSW, Dimitriadis S, Mark WA. Examining links between anxiety, reinvestment and walking when talking by older adults during adaptive gait. Exp Brain Res 2016; 234: 161–72. 26403296 10.1007/s00221-015-4445-z PMC4713710

[58] Clark DJ, Rose DK, Ring SA, Porges EC. Utilization of central nervous system resources for preparation and performance of complex walking tasks in older adults. Front Aging Neurosci 2014; 6: 217. 10.3389/fnagi.2014.00217. 25202270 PMC4142860

[59] Clark DJ . Automaticity of walking: functional significance, mechanisms, measurement and rehabilitation strategies. Front Hum Neurosci 2015; 9: 1–13. 25999838 10.3389/fnhum.2015.00246 PMC4419715

[60] Reynolds RF, Day BL. Visual guidance of the human foot during a step. J Physiol 2005; 569: 677–84. 16179363 10.1113/jphysiol.2005.095869 PMC1464243

[61] Brown LA, Doan JB, McKenzie NC, Cooper SA. Anxiety-mediated gait adaptations reduce errors of obstacle negotiation among younger and older adults: implications for fall risk. Gait Posture 2006; 24: 418–23. 16420978 10.1016/j.gaitpost.2005.09.013

[62] Bermúdez JP . Do we reflect while performing skillful actions? Automaticity, control, and the perils of distraction. Philosoph Psych 2017; 30: 896–924.

[63] Johnson KJ, Watson AM, Tokuno CD, Carpenter MG, Adkin AL. The effects of distraction on threat-related changes in standing balance control. Neurosci Lett 2020; 716: 134635. 10.1016/j.neulet.2019.134635. 31751670

[64] Baumeister RF, Masicampo EJ, Vohs KD. Do conscious thoughts cause behavior? Annu Rev Psychol 2011; 62: 331–61. 21126180 10.1146/annurev.psych.093008.131126

[65] Aizen E . Cautious gait and fear of falling in the elderly. Harefuah 2001; 140: 1091–4, 1115. 11759389

[66] Herman T, Giladi N, Gurevich T, Hausdorff JM. Gait instability and fractal dynamics of older adults with a ‘cautious’ gait: why do certain older adults walk fearfully? Gait Posture 2005; 21: 178–85. 15639397 10.1016/j.gaitpost.2004.01.014

[67] Parry SW, Bamford C, Deary V et al. Cognitive-behavioural therapy-based intervention to reduce fear of falling in older people: therapy development and randomised controlled trial—the strategies for increasing independence, confidence and energy (STRIDE) study. Health Technol Assess 2016; 20: 1–206. 10.3310/hta20560 PMC4983706 27480813

[68] Ellmers TJ, Cocks AJ, Young WR. Exploring attentional focus of older adult fallers during heightened postural threat. Psychol Res 2020; 84: 1877–89. 31119367 10.1007/s00426-019-01190-6 PMC7479009

[69] Adamczewska N, Nyman SR. A new approach to fear of falls from connections with the posttraumatic stress disorder literature. Gerontol Geriatr Med 2018; 4: 233372141879623. 10.1177/2333721418796238. PMC6111705 30182037

[70] Eysenck MW, Derakshan N, Santos R, Calvo MG. Anxiety and cognitive performance: attentional control theory. Emotion 2007; 7: 336–53. 17516812 10.1037/1528-3542.7.2.336

[71] Hadjistavropoulos T, Carleton NR, Delbaere K et al. The relationship of fear of falling and balance confidence with balance and dual tasking performance. Psychol Aging 2012; 27: 1–13. 21707181 10.1037/a0024054

[72] Nagamatsu LS, Voss M, Neider MB et al. Increased cognitive load leads to impaired mobility decisions in seniors at risk for falls. Psychol Aging 2011; 26: 253–9. 21463063 10.1037/a0022929 PMC3123036

[73] Monaghan AS, Johansson H, Torres A, Brewer GA, Peterson DS. The impact of divided attention on automatic postural responses: a systematic review and meta-analysis. Exp Gerontol 2022; 162: 111759. 10.1016/j.exger.2022.111759. 35245641

[74] Matthis JS, Barton SL, Fajen BR. The critical phase for visual control of human walking over complex terrain. Proc Natl Acad Sci U S A 2017; 114: E6720–9. 28739912 10.1073/pnas.1611699114 PMC5558990

[75] Patla AE, Vickers JN. Where and when do we look as we approach and step over an obstacle in the travel path? Neuroreport 1997; 8: 3661–5. 9427347 10.1097/00001756-199712010-00002

[76] Ellmers TJ, Young WR. The influence of anxiety and attentional focus on visual search during adaptive gait. J Exp Psychol Hum Percept Perform 2019; 45: 697–714. 31120299 10.1037/xhp0000615

[77] Young WR, Hollands MA. Can telling older adults where to look reduce falls? Evidence for a causal link between inappropriate visual sampling and suboptimal stepping performance. Exp Brain Res 2010; 204: 103–13. 20512484 10.1007/s00221-010-2300-9

[78] Young WR, Ellmers TJ. Translating attentional control theory to applied psychological eye tracking research. In: Stuart S, ed. Eye Tracking: Background, Methods, and Applications. Humana, New York, NY, 2022: 131–49.

[79] Rasmussen B, Nielsen CV, Uhrenfeldt L. Being active after hip fracture; older people’s lived experiences of facilitators and barriers. Int J Qual Stud Health Well Being 2018; 13: 13. 10.1080/17482631.2018.1554024. PMC6327563 30704373

[80] Delbaere K, Crombez G, Van Haastregt JCM, Vlaeyen JWS. Falls and catastrophic thoughts about falls predict mobility restriction in community-dwelling older people: a structural equation modelling approach. Aging Ment Heal 2009; 13: 587–92. 10.1080/13607860902774444 19629784

[81] Rider JV, Longhurst JK, Lekhak N, Navalta JW, Young DL, Landers MR. Psychological factors associated with fear of falling avoidance behavior in Parkinson’s disease: the role of depression, anxiety, and catastrophizing. J Geriatr Psychiatry Neurol 2022; 36: 215–24. 35977708 10.1177/08919887221119974

[82] Lawrence RH, Tennstedt SL, Kasten LE, Shih J, Howland J, Jette AM. Intensity and correlates of fear of falling and hurting oneself in the next year: baseline findings from a Roybal Center fear of falling intervention. J Aging Health 1998; 10: 267–86. 10342933 10.1177/089826439801000301

[83] Sturnieks DL, Delbaere K, Brodie MA, Lord SR. The influence of age, anxiety and concern about falling on postural sway when standing at an elevated level. Hum Mov Sci 2016; 49: 206–15. 27428596 10.1016/j.humov.2016.06.014

[84] Mokkink LB, Terwee CB, Patrick DL et al. The COSMIN checklist for assessing the methodological quality of studies on measurement properties of health status measurement instruments: an international Delphi study. Qual Life Res 2010; 19: 539–49. 20169472 10.1007/s11136-010-9606-8 PMC2852520

[85] Kempen G, Yardley L, Van Haastregt JCM et al. The short FES-I: a shortened version of the falls efficacy scale-international to assess fear of falling. Age Ageing 2008; 37: 45–50. 18032400 10.1093/ageing/afm157

[86] Zigmond AS, Snaith RP. The hospital anxiety and depression scale. Acta Psychiatr Scand 1983; 67: 361–70. 6880820 10.1111/j.1600-0447.1983.tb09716.x

[87] Zijlstra GAR, Van Haastregt JCM, Ambergen T et al. Effects of a multicomponent cognitive behavioral group intervention on fear of falling and activity avoidance in community-dwelling older adults: results of a randomized controlled trial. J Am Geriatr Soc 2009; 57: 2020–8. 19793161 10.1111/j.1532-5415.2009.02489.x

[88] Adkin AL, Frank JS, Carpenter MG, Peysar GW. Postural control is scaled to level of postural threat. Gait Posture 2000; 12: 87–93. 10998604 10.1016/s0966-6362(00)00057-6

[89] Johnson KJ, Zaback M, Tokuno CD, Carpenter MG, Adkin AL. Exploring the relationship between threat-related changes in anxiety, attention focus, and postural control. Psychol Res 2019; 83: 445–58. 29110077 10.1007/s00426-017-0940-0

[90] Zaback M, Adkin AL, Chua R, Inglis JT, Carpenter MG. Facilitation and habituation of cortical and subcortical control of standing balance following repeated exposure to a height-related postural threat. Neuroscience 2022; 487: 8–25. 35085706 10.1016/j.neuroscience.2022.01.012

[91] Brown LA, Polych MA, Doan JB. The effect of anxiety on the regulation of upright standing among younger and older adults. Gait Posture 2006; 24: 397–405. 17055728 10.1016/j.gaitpost.2005.04.013

[92] McKenzie NC, Brown LA. Obstacle negotiation kinematics: age-dependent effects of postural threat. Gait Posture 2004; 19: 226–34. 15125911 10.1016/S0966-6362(03)00060-2

[93] Brown LA, Gage WH, Polych MA, Sleik RJ, Winder TR. Central set influences on gait. Age-dependent effects of postural threat. Exp Brain Res 2002; 145: 286–96. 12136378 10.1007/s00221-002-1082-0

[94] Caetano MJD, Gobbi LTB, Sánchez-Arias M d R, Stella F, Gobbi S. Effects of postural threat on walking features of Parkinson’s disease patients. Neurosci Lett 2009; 452: 136–40. 19383427 10.1016/j.neulet.2009.01.053

[95] Tersteeg MCA, Marple-Horvat DE, Loram ID. Cautious gait in relation to knowledge and vision of height: is altered visual information the dominant influence? J Neurophysiol 2012; 107: 2686–91. 22378173 10.1152/jn.00875.2011

[96] Brown LA, Sleik RJ, Polych MA, Gage WH. Is the prioritization of postural control altered in conditions of postural threat in younger and older adults? J Gerontol A Biol Sci Med Sci 2002; 57: M785–92. 12456737 10.1093/gerona/57.12.m785

