# Supplementary material for: The perceived control model of falling: developing a unified framework to understand and assess maladaptive fear of falling
Source: Age Ageing. 2023 Jul 15;52(7):afad093. doi: 10.1093/ageing/afad093 (PMC10355179; doi:10.1093/ageing/afad093)
Supplement: Supplementary_Materials_(1)_afad093 [file supplementary_materials_(1)_afad093.pdf]

1. Participants

Eligibility and recruitment strategy. Participants were eligible for participation if they were ≥ 60 years of age and if they had not previously been diagnosed with dementia or any other degenerative neurological disease. Participants were recruited from community social networks, exercise classes and older adult research participation groups (such as the Brunel Older Adult Reference Group; University of the 3rd age), through advertisements sent out in newsletters to members and/or placed on online noticeboards. The advertisements contained a brief description of the research, and a link to a webpage that housed a longer description, the participant information sheet, the consent form, and the survey itself.

Sample size. With reference to sample size, main analyses of interest were exploratory factor analysis, and retest-reliability analysis. For the factor analysis we aimed for a sample of 200 older adults. For the test-retest reliability we aimed for 60 individuals with complete data for the newly developed UP-COF questionnaire at timepoint 1 and timepoint 2 (T1 and T2). That would ensure 80% power to detect an intraclass correlation coefficient of .80 (95%CI: .70-.90).

Flow of the study and participant characteristics. In total, 213 participants completed the study at T1, of which 4 were excluded due to having been diagnosed with Parkinson's Disease (N=3) or participating twice (N=1; second entry excluded from analysis). Participant characteristics of the remaining 209 participants are summarised in Table S1.

Table S1. *Participant Characteristics.*

	**Older adults (N = 209)**
**Background / General Health**	
Age in years *(mean ± SD [range])*	75.5 ± 6.9 [60-94]
Female gender *(n; %)*	170 (81.3%)
Education Level	
Prefer not to say / unknown	6 (3%)
High School	26 (12%)
College / sixth form	45 (22%)
University – undergraduate	57 (27%)
University – postgraduate	75 (36%)
General health < Good *(n; %)*	22 (11%) [1 missing]
Medication use >4 *(n; %)*	32 (15%) [2 missing]
Peripheral neuropathy *(n; %)*	11 (5%) [2 missing]
Diabetes *(n; %)*	19 (9%) [2 missing]
**Physical functioning**	
Falls in past 12 months *(n; %)*	69 (33%) [2 missing]
Self-reported balance problems *(n; %)*	82 (40%) [2 missing]
Walking aid *(n; %)*[3]	30 (15%) [4 missing]
Dizziness in daily life[x] *(n; %)*	68 (33%) [1 missing]
**Psychological functioning**	
Self-reported fear of falling [2 missing]	
Not at all	41 (20%)
A little	134 (65%)
Quite a bit	24 (12%)
Very much	8 (4%)
Self-reported activity avoidance due to fear of falling	61 (30%) [3 missing]
Short Falls Efficacy Scale-International (7-28; *mean±SD [range])*	10.1 ± 3.3 [7-25] [1 missing]
Hospital Anxiety and Depression Scale-Anxiety (0-21) *(mean±SD [range])*	4.4 ± 3.6 [0-17] [1 missing]
Depression Diagnosis *(n; %)*	39 (19%) [2 missing]

2. Overview of Procedures

At T1, participants completed the newly developed 6-item UP-COF, along with questions on background characteristics, and the anxiety subscale of the Hospital Anxiety Depression Scale (HADS) and the short Falls-Efficacy Scale-International (FES-I), measures of generalised anxiety and concerns about falling, respectively. These measures were collected to be able to test whether the UP-COF scale scores would be associated with these measures, which is a measure of construct validity (as all measures should be tapping into the same construct to some degree, they should show moderate correlations). To assess re-test reliability, a subset (N=65) of participants were invited to complete the UP-COF again 2 weeks after T1 (mean re-test interval: 16.2 days, SD = 5.1, range: 6-26 days). In total, test-retest data was available for 57 participants (female = 80.7%, M age = 77.7, SD = 6.6).

The UP-COF scale then was validated in a step-wise fashion, outlined below. Following recommendations [1], we first evaluated the scale at item-level, performed explorative factor analysis, and assessed test-retest reliability and concurrent validity of total UP-COF scores, as well as evaluated measurement error and minimal detectable change. Finally, we conducted ROC analyses to determine relevant cut-offs.

ROC analyses were conducted to determine optimal cut-off points to determine: (1) repeated fallers vs. non-fallers; (2) individuals reporting fear-related activity restriction vs. no activity restriction; (3) individuals reporting dizziness vs. no dizziness, and; (4) individuals meeting the cut-off point for anxiety using the HADS (≥8/21) vs. below this cut-off value.

We discuss and present results for each steps below in turn.

3. Validation steps and results

Step 1. Evaluation of scale at item-level based on completeness, score range, and re-test reliability.

Table S2 summarises the results of initial evaluation of the UP-COF scale's items, based on the T1 data for the entire sample (N=209). There were no issues noted with missing data, as there were no missing cases for any of the items. Test-retest reliability (2-way, random effect, consistency single measures Intraclass Correlation Coefficient) indices were sufficient to good (.5-.7) for all items except for item 2 (.089), which had really poor retest reliability. This item was therefore removed from further analysis. Item 4's ICC value was just below the recommended threshold (.447 [2]). Regarding score range/distribution, items 5 and 6 had relatively many individuals scoring the maximal value (~60%). Discussion among the research team led to the decision to exclude item 2, but to keep items 4-6 for use in the next step – the exploratory factor analysis.

Table S2. *Results of initial evaluation of the UP-COF items.*

Item	*n / % missing*	*% min/max score*	*ICC (95% CI)*	*Included in next analysis step?*
1. I can reduce my risk of falling	0 / 0%	1% / 31%	.566 (.360, .719)	Yes
2. I can overcome any fear of falling I experience	0 / 0%	2% / 28%	.089 (-.173, .340)	No
3. I can easily put worries about falling out of my mind	0 / 0%	3% / 30%	.726 (.575, .829)	Yes
4. There are things I can do to keep myself from falling	0 / 0%	1% / 37%	.447 (.213, .632)	Yes
5. I find myself panicking about falling	0 / 0%	2% / 62%	.502 (.280, .673)	Yes
6. I can stop fear of falling from overwhelming me	0 / 0%	1% / 61%	.539 (.326, .700)	Yes

NB: Predetermined cut-off values were 5% (missing cases per item), 50% (% of maximal / minimal scores for an item), and ICC<.500. Excluded item highlighted in red.

Step 2. Exploratory Factor Analysis

First, we assessed the inter-item correlations for the remaining items. These ranged from r=.218 to r=.537, and were therefore acceptable. Exploratory factor analysis using maximal likelihood estimation showed that all items loaded onto one underlying factor. However, as item 5's factor loading was close to meeting the cut-off threshold (.400 [3]), and combined with the earlier reported tendency toward a ceiling effect for that item, we decided to remove it from the analysis. The second run of the factor analysis found satisfactory factor loadings for all remaining items, and confirmed unidimensionality of the scale. Table S3 summarised the key results of this analysis.

Table S3. *Factor loadings for each item, presented separately for each of the two runs of the factor analysis.*

**Item**	**RUN 1 [a]**	**RUN 2 [b] (after excluding Item 5)**
	Component Loading (explained variance 51.4%) *Component 1*	Component Loading (explained variance 58.2%) *Component 1*
1. I can reduce my risk of falling	**.577**	**.620**
3. I can easily put worries about falling out of my mind	**.675**	**.629**
4. There are things I can do to keep myself from falling	**.680**	**.738**
5. I find myself panicking about falling	**.471**	N/A
6. I can stop fear of falling from overwhelming me	**.721**	**.674**

[a] Kaiser-Meyer-Olkin assessment (KMO)=.766; all individual KMOs≥.738 (>0.5 threshold).
[b] KMO=.726; individual KMOs≥.718; Excluded item highlighted in red.

Step 3. Reliability, Measurement Error, and Validity of Total Scores on Final UP-COF

Average total score on the final 4-item UP-COF scale at T1 was 15.9 (SD=3.2) for the entire sample (N=209). Based on the data of all participants at T1, the internal consistency of the UP-COF scale was good (Cronbach's alpha = .751).

For those 57 participants who had been included in the test-retest analysis, UP-COF scores were 16.2 (SD=2.7) at T1 and 16.6 (SD=2.9) at T2. Test-retest reliability was good to excellent (ICC = .718).

Regarding measurement error, the standard error of the measurement (SEM) was 1.5, while the minimal detectable difference was 0.54 on group level and 4.1 on individual level.

Regarding validity, the total UP-COF scores at T1 were significantly negatively correlated with FES-I scores (*r*=-.567, 95%CI=[-.653, -.467], *p*<.001) and HADS-anxiety scores (*r*=-.410, 95%CI=[-.518, -.291], *p*<.001).

UP-COF scores were significantly lower in individuals who had fallen repeatedly in the past 12 months (*M*=12.9, *SD*=4.68) compared to both non-fallers (*M*=16.4, *SD*=2.84, *Z*=-3.65, *p*<.001) and those who had fallen once (*M*=16.2, *SD*=2.61, *Z*=-3.06, *p*=.002). Scores were also significantly lower in: individuals who reported that they avoided activities due to fear of falling (*M*=14.0, *SD*=3.75), compared to those who did not (*M*=16.7, *SD*=2.60, *Z*=-5.26, *p*<.001); those who reported dizziness (*M*=14.9, *SD*=3.66) versus those who did not (*M*=16.5, *SD*=2.86, *Z*=-3.30, *p*=.001), and; those scoring above (*M*=13.4, *SD*=4.2) versus below the cut-off for anxiety, using the HADS (*M*=16.5, *SD*=2.7, *Z*=-4.45, *p*<.001).

Step 4. ROC analyses

Area under the curve scores were 0.74 for repeated fallers vs. non-fallers; 0.73 for individuals reporting fear-related activity restriction vs. no activity restriction; 0.64 for individuals reporting dizziness vs. no dizziness, and; 0.73 for individuals meeting the cut-off point for anxiety using the HADS vs. below this cut-off value.

Based on these analyses, we defined the cut of point of ≤13/20 on the UP-COF to identify someone as having low perceived control of falling. 21.2% of our sample who reported experiencing fear of falling to some degree in daily life met this threshold (see Figure S1 below).

Figure S1. *Distribution of total UP-COF scores with respect to self-reported fear of falling, and the ROC cut-off for low perceived control of falling (≤13/20; participants in red box).*

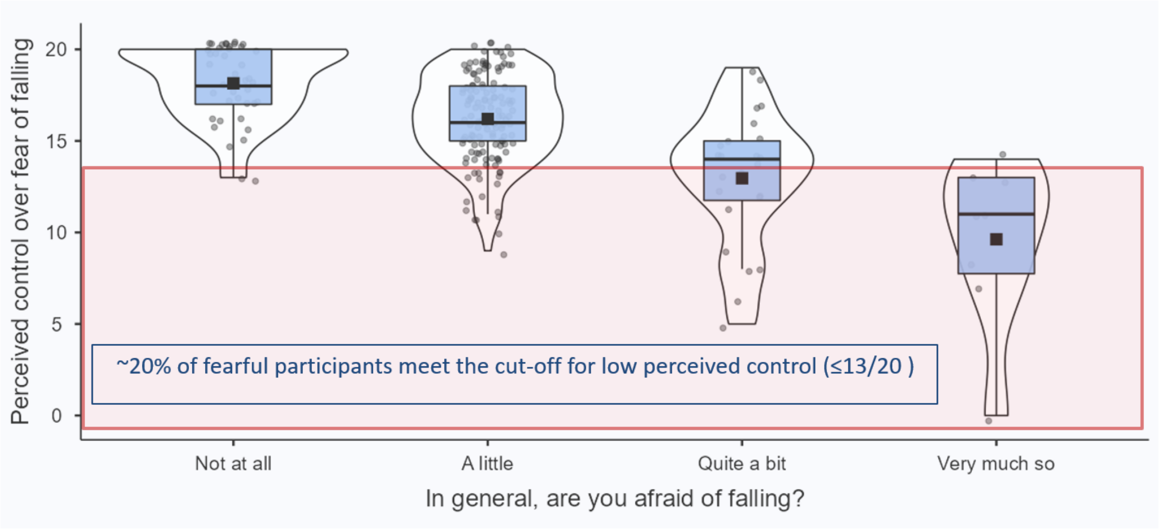

References

[1] L.B. Mokkink, C.B. Terwee, D.L. Patrick, J. Alonso, P.W. Stratford, D.L. Knol, L.M. Bouter, H.C.W. De Vet, The COSMIN checklist for assessing the methodological quality of studies on measurement properties of health status measurement instruments: An international Delphi study, Qual. Life Res. 19 (2010) 539–549. https://doi.org/10.1007/s11136-010-9606-8.

[2] L.G. Portney, M.P. Watkins, Foundations of Clinical Research: Applications to Practice Hardcover, Pearson Education, Harlow, UK, 2007.

[3] J.P. Stevens, Applied multivariate statistics for the social sciences, Erlbaum, Hillsdale, New York, 2002.
