# Supplementary material for: The perceived control model of falling: developing a unified framework to understand and assess maladaptive fear of falling
Source: Age Ageing. 2023 Jul 15;52(7):afad093. doi: 10.1093/ageing/afad093 (PMC10355179; doi:10.1093/ageing/afad093)
Supplement: aa-22-1885-File003_afad093 [file aa-22-1885-file003_afad093.pdf]

# Updated Perceived Control over Falling Scale (UP-COF)

Please answer the below questions with respect to how you have *generally* felt in **recent weeks**.

**I can reduce my risk of falling**

Strongly disagree 0 1 2 3 4 5 strongly agree

**I can easily put worries about falling out of my mind**

Strongly disagree 0 1 2 3 4 5 strongly agree

**There are things I can do to keep myself from falling**

Strongly disagree 0 1 2 3 4 5 strongly agree

**I can stop fear of falling from overwhelming me**

Strongly disagree 0 1 2 3 4 5 strongly agree

**Total Score: __________**

A score of 13/20 or lower indicates low perceived control.

Ellmers, T.J., Wilson, M.R., Kal, E.C., & Young, W.R. (2023)

**Contact for permissions:** t.ellmers@imperial.ac.uk
